# Alkylation Base Damage Is Converted into Repairable Double-Strand Breaks and Complex Intermediates in G2 Cells Lacking AP Endonuclease

**DOI:** 10.1371/journal.pgen.1002059

**Published:** 2011-04-28

**Authors:** Wenjian Ma, Jim W. Westmoreland, Dmitry A. Gordenin, Mike A. Resnick

**Affiliations:** Chromosome Stability Group, Laboratory of Molecular Genetics, National Institute of Environmental Health Sciences (NIEHS), National Institutes of Health, Research Triangle Park, North Carolina, United States of America; University of Washington, United States of America

## Abstract

DNA double-strand breaks (DSBs) are potent sources of genome instability. While there is considerable genetic and molecular information about the disposition of direct DSBs and breaks that arise during replication, relatively little is known about DSBs derived during processing of single-strand lesions, especially for the case of single-strand breaks (SSBs) with 3′-blocked termini generated *in vivo*. Using our recently developed assay for detecting end-processing at *random* DSBs in budding yeast, we show that single-strand lesions produced by the alkylating agent methyl methanesulfonate (MMS) can generate DSBs in G2-arrested cells, *i.e.*, S-phase independent. These derived DSBs were observed in *apn1/2* endonuclease mutants and resulted from aborted base excision repair leading to 3′ blocked single-strand breaks following the creation of abasic (AP) sites. DSB formation was reduced by additional mutations that affect processing of AP sites including *ntg1*, *ntg2*, and, unexpectedly, *ogg1*, or by a lack of AP sites due to deletion of the *MAG1* glycosylase gene. Similar to direct DSBs, the derived DSBs were subject to MRX (Mre11, Rad50, Xrs2)-determined resection and relied upon the recombinational repair genes *RAD51*, *RAD52*, as well as on the *MCD1* cohesin gene, for repair. In addition, we identified a novel DNA intermediate, detected as slow-moving chromosomal DNA (SMD) in pulsed field electrophoresis gels shortly after MMS exposure in *apn1/2* cells. The SMD requires nicked AP sites, but is independent of resection/recombination processes, suggesting that it is a novel structure generated during processing of 3′-blocked SSBs. Collectively, this study provides new insights into the potential consequences of alkylation base damage *in vivo*, including creation of novel structures as well as generation and repair of DSBs in nonreplicating cells.

## Author Summary

DNA double-strand breaks (DSBs) are an important source of genome instability that can lead to severe biological consequences including tumorigenesis and cell death. Although much is known about DSBs induced directly by ionizing radiation and radiomimetic cancer drugs, there is a relative dearth of information about the formation of derived DSBs that arise from processing of single-strand lesions. Since as many as 10,000–200,000 single-strand lesions have been estimated to occur each day in mammalian cells, conversion of even a small percentage of such lesions to DSBs could dramatically affect genome stability. Here we addressed the mechanism of formation and repair of derived DSBs *in vivo* during the processing of DNA methylation damage in yeast that are defective in base excision repair (BER) due to a lack of AP endonucleases. Armed with a technique developed in our lab that detects resection at DSBs, a first step in DSB repair, we demonstrated formation of DSBs in G2 cells and the role of recombinational repair in subsequent chromosome restitution. Furthermore, we have identified a novel repair intermediate that can be generated if abasic sites are nicked by AP lyases, providing additional insights into the processing of 3′-blocked groups at single-strand breaks.

## Introduction

DNA double-strand breaks (DSBs) are important sources of genome instability, giving rise to chromosomal aberrations and severe biological consequences including tumorigenesis and cell death [1], [2]. We and others also showed that regions adjacent to DSBs are prone to mutagenesis through a variety of mechanisms [3]–[7]. DSBs can be induced directly by exposure to DNA-damaging agents such as ionizing radiation (IR) and radiomimetic chemicals. While there is a great deal of information about direct DSBs, little is known about the contribution of single-strand lesions to the production of DSBs, although single-strand lesions are generally accepted to be a source of DSBs via replication fork collapse in regions of single-strand DNA [8].

A common single-strand lesion that is generated during normal cell metabolism and repair is an apurinic/apyrimidic (AP) site, one of the most abundant DNA lesions in the cell [9], [10]. As many as 10,000–200,000 single-strand lesions appear each day in mammalian cells [11], [12]. Most of these are subject to base excision repair (BER), a highly coordinated process initiated by a lesion-specific glycosylase removing damaged bases and forming AP sites. Removal of AP sites by AP endonucleases or AP lyases involves the generation of single-strand breaks (SSBs) with blocking groups at their 3′ or 5′-ends that cannot be joined by DNA ligases [13]–[15]. Subsequent SSB end-processing involves a diverse set of enzymes/pathways to deal with the termini [16].

Single strand lesions which are produced by many mutagens are also potential sources of DSBs if they are processed to form closely-opposed SSBs. Closely-opposed SSBs could result in derived DSBs simply through loss of pairing of short DNA duplex regions bounded by the SSBs, as shown by *in vitro* analysis [17]–[19] and a limited number of *in vivo* studies [20], [21]. A DSB could also be generated if two more distant SSBs are processed to form closely-opposed SSBs. This second category of derived DSBs have been proposed following induction of methyl methanesulfonate (MMS) lesions and subsequent processing of AP sites to 5′-blocked SSB termini in *rad27/FEN1* and *pol32* mutants [20]. Removal of these 5′-blocked SSB ends involves DNA synthesis and strand displacement that can move distant SSBs closer [20], [22]. However, there is little information about *in vivo* generation of derived DSBs from nearby opposed SSBs with 3′-blocked termini. Such termini are a challenge to the repair machinery since they must be removed to enable repair synthesis at 3′-OH ends [23], [24]. Besides being formed directly from sugar damage, SSBs with 3′-blocked termini, the α,β-unsaturated aldehyde (3′-dRP), can be generated during incision at the 3′-side of AP sites by AP lyase [14].

In the budding yeast *Saccharomyces cerevisiae*, the AP endonucleases Apn1 and Apn2, which have 3′-phosphodiesterase activity, are responsible for removing most 3′-dRP ends as well as other blocking groups [25]–[27]. Previously, we found that deletion of both AP endonucleases appears to lead to accumulation of chromosome breaks in nongrowing G1 haploid yeast [28] and the number of chromosome breaks increased with time of liquid-holding in buffer. However, it was possible that the DSBs were not formed *in vivo* but actually appeared during subsequent pulsed-field gel electrophoresis (PFGE) processing. Therefore, while those findings highlighted the potential for single-strand damage to generate DSBs, they did not definitively show that the derived DSBs were generated *in vivo* or that they could be generated later in the cell cycle. Importantly, there was no evidence of repair of the DSBs, which is not surprising since the haploid cells were in the G1 phase of the cell cycle when there would be no recombinational partner. Our previous study also showed that non-homologues recombination (NHEJ) has little if any role in dealing with the derived “DSBs” caused by MMS in G1 cells based on deletion of yku70, especially for the *apn1/2* mutant which accumulated DSBs even though it has the wild type NHEJ machinery [28].

Although there is abundant genetic evidence for homologous recombinational (HR) repair proteins dealing with MMS damage-induced lesions in a variety of systems, there has been no direct demonstration of MMS-induced DSBs being formed or subsequent repair *in vivo*. It is generally assumed, though not proven, that recombinational repair deals with DSBs generated during replication fork collapse following induction of MMS lesions. Here, we demonstrate MMS can generate derived DSBs within G2/M arrested cells and that these DSBs are processed and undergo repair. Utilizing our recently developed PFGE assay [29], we establish that MMS-derived DSB ends are subject to resection, one of the earliest steps in DSB repair. In addition we identify a novel repair intermediate detected as slow mobility chromosomal DNA during PFGE, providing additional insights into the processing of 3′-blocked groups *in vivo*.

## Results

### Generation and repair of DSBs in G2 cells that lack the Apn1 Apn2 endonucleases

We previously described a system using PFGE for analyzing *in vivo* repair of alkylation base damage caused by MMS [28] in yeast that is based on detection of chromosome breaks. Though MMS does not cause SSBs directly [28], [30], they can arise as repair intermediates during BER. If the SSBs are closely-spaced on complementary DNA strands, they are detected as “DSBs” with PFGE. Most of the closely-opposed single strand lesions were shown to be efficiently repaired in stationary G1 haploid wild type cells by BER [28], thereby preventing the formation of derived DSBs *in vivo*. We now extend this system to a characterization of derived DSBs in G2 cells where there is the opportunity for recombinational repair between sister chromatids.

**Figure 1:**
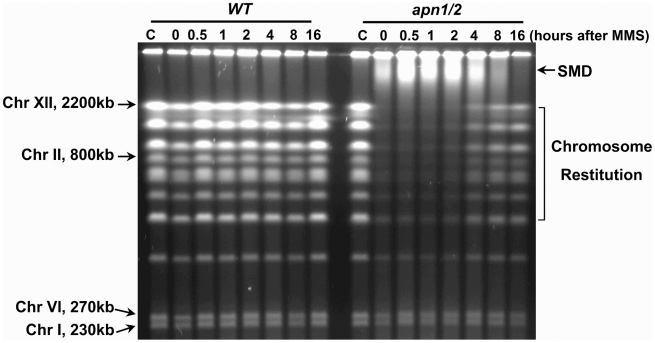
MMS–induced chromosomal damage, SMD, and repair in G2 yeast. WT and *apn1/2* haploid strains growing in YPDA were arrested at G2/M by nocodazole and treated with 0.1% (11.8 mM) MMS for 15 min in PBS. Cells were returned to YPDA containing nocodazole and incubated for up to 16 hours (30°C). Cells were collected and processed for PFGE analysis. Following PFGE, chromosomes were visualized by ethidium bromide staining. The letter “C” above the first lane stands for mock-treated control samples (the same applies to the rest of the figures). The slow moving DNA (SMD) in the *apn1/2* mutant is detected as a wide band of DNA between the largest chromosome (Chr XII) and the wells following treatment.

**Figure 2:**
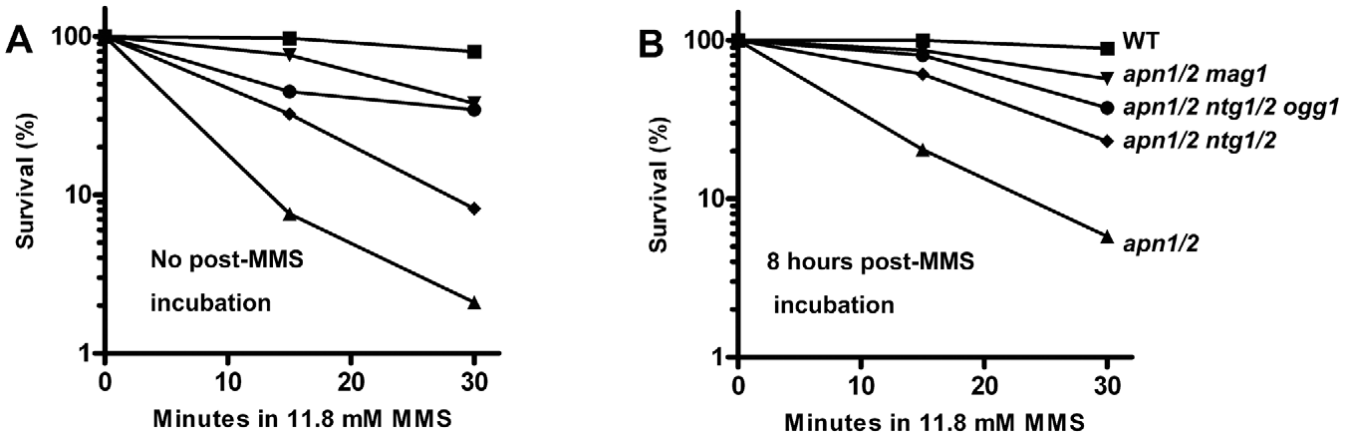
MMS–induced killing of WT and various *apn1, 2* derived mutants. Logarithmically growing cells were arrested in G2/M with nocodazole, treated with MMS in PBS (0.1%, 15 min, 30 min) and returned to the YPDA+nocodazole medium for post-MMS incubation for 8 hours. The cell survivals of MMS-treated cells, without (A) and with (B) post-MMS incubation were done by plating cells to YPDA plates. The percentage is the ratio of colonies arising after MMS treatment vs mock treatment (done in triplicate). WT (▪), *apn1/2* (▴), *apn1/2 mag1*(▾), *apn1/2 ntg1/2* (♦) and *apn1/2 ntg1/2 ogg1* (•).

Haploid yeast were grown to log phase in rich medium (YPDA), arrested in G2/M with the microtubule and mitotic spindle disrupter drug nocodazole and treated with 0.1% MMS (11.8 mM) for 15 min in PBS. They were subsequently incubated in YPDA+nocodazole to prevent G2 cells from progression into the next cell cycle stage. Changes in chromosomes at various times after treatment were determined using PFGE. The treatment of WT cells with MMS did not cause fragmentation of chromosomes (which range in size from ∼200 kb to ∼2.5 Mb; Figure 1), suggesting that there is efficient repair of single strand damage and, therefore, no apparent generation of DSBs. There was also no apparent reduction in survival (Figure 2). However, MMS treatment of *apn1 apn2* (*apn1/2*) cells led to loss of all but the smaller chromosome bands (Chr I, 230 kb and Chr VI, 270 kb) as well as decreased survival (Figure 2). At later times, there is “restitution” of the broken chromosomes *(i.e.*, formation of full size chromosomes) as shown in the ethidium bromide stained gel (indicated in Figure 1), and the survival is somewhat higher for MMS-treated *apn1/2* after 8 hour G2/M holding. Surprisingly, there was also a rapid accumulation of slow-moving DNA (SMD) that appeared below the well. These results differ from those with MMS-treated stationary arrested G1 *apn1/2* cells which did not give rise to SMD although chromosome breakage was detected by PFGE [28]. The amount of SMD decreased after 4 hours, at which time restituted chromosomes were detected. The mechanism(s) of induction and disappearance of DSBs and SMD, as well as possible relationship, is investigated below.

### Deletion of *MAG1* or stabilization of AP sites prevents DSBs and SMD

**Figure 3:**
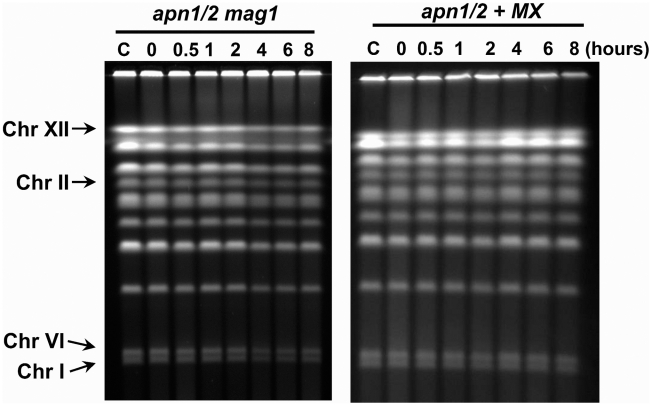
MMS–derived DSBs and SMD require abasic site (AP) intermediates and are prevented by a *mag1 deletion* mutation or methoxyamine (MX). The logarithmically growing *apn1/2* and *apn1/2 mag1* cells in YPDA were arrested at G2/M by nocodazole, treated with MMS (0.1%, 15 min) in PBS and returned to YPDA+nocodazole and incubated further. The cells were incubated with MX (100 mM) prior to, during and following MMS treatment. Cells were collected at the indicated times and processed for PFGE analysis.

In yeast, the first step in the BER of MMS-induced lesions requires Mag1 glycosylase which removes damaged bases and forms abasic sites [31]. To confirm that the DSBs as well as SMD resulted from BER, the *MAG1* gene was deleted in the *apn1/2* background. As shown in Figure 3, the appearance of DSBs and SMD requires at least the first step in BER in G2 arrested cells since there was no apparent change in chromosomes and no SMD formation in the triple *apn1/2 mag1* mutants following MMS treatment. Although methylated bases can be spontaneously depurinated to form AP sites, the impact of this process to DSB formation is limited as indicated by the limited appearance of DSBs in the *apn1/2 mag1* mutant (Figure S3). The formation of AP sites and subsequent DSBs were also prevented in the triple mutant arrested as G1 stationary cells [28]. The absence of Mag1 also resulted in a considerable increase in toleration of MMS damage (Figure 2). We examined further the role of AP sites by including methoxyamine (MX) during the MMS treatment and subsequent incubation of the *apn1/2*. Methoxyamine covalently binds to AP sites, preventing subsequent BER processing [32]. The MX results shown in Figure 3 were similar to those observed with the *mag1 apn1/2* mutant. Thus, the appearance of DSBs and SMD in the G2/M cells lacking the Apn1 and Apn2 endonucleases, along with increased MMS hypersensitivity, requires the generation of AP sites by BER and/or repair events downstream of AP sites.

### Homologous recombination (HR) defects impede chromosome restitution but do not affect SMD

**Figure 4:**
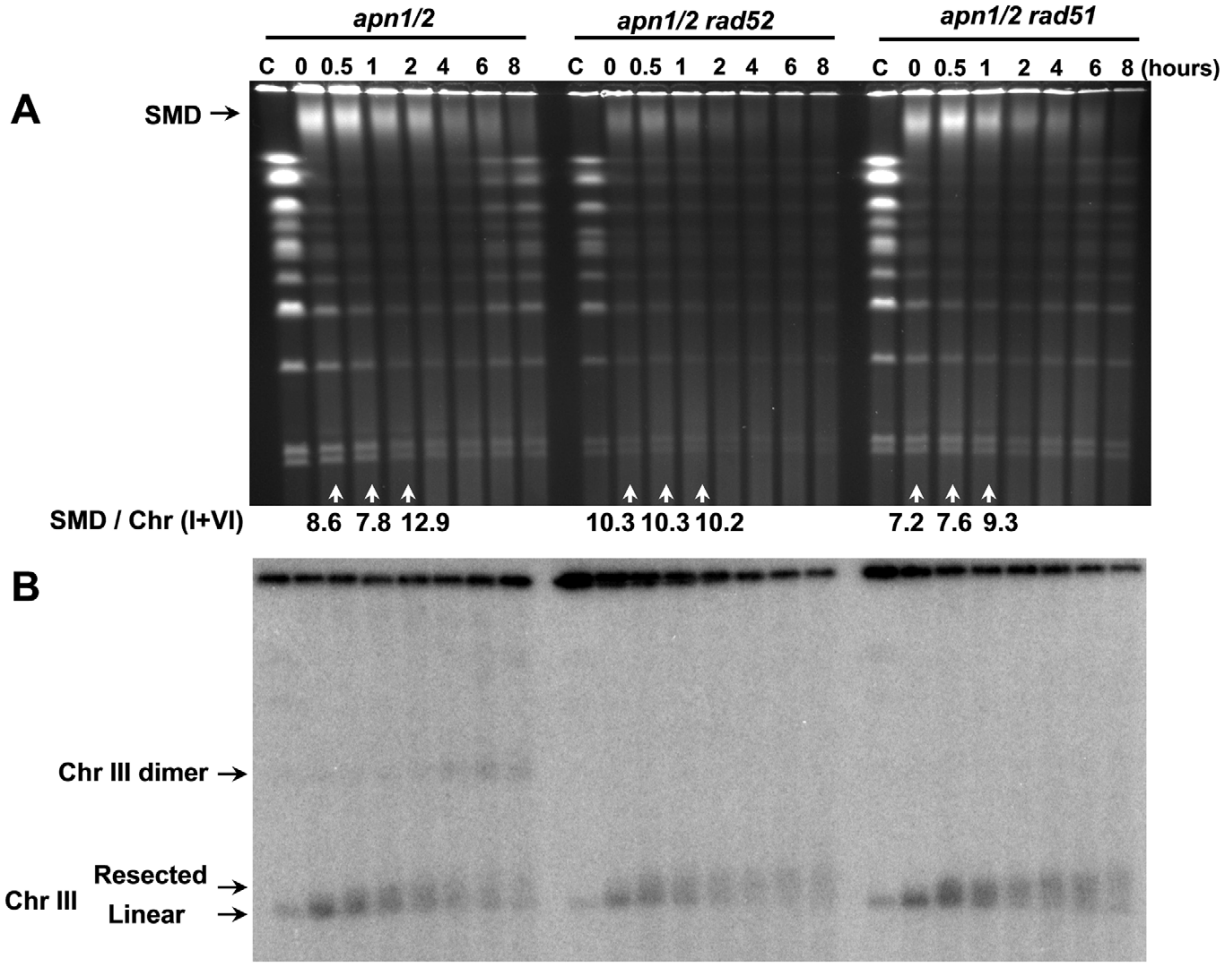
Resection, recombinants, and HR–dependent restitution of MMS–derived DSBs. Logarithmically growing *apn1/2* cells as well as triple mutants containing a deletion of an HR gene *rad52* or *rad51* were arrested in G2/M with nocodazole, treated with MMS (0.1%, 15 min) in PBS and returned to the YPDA+nocodazole medium. Cells were collected at the indicated times and processed for PFGE analysis. Chromosomes were visualized by ethidium bromide staining (A) and by Southern blotting (B) with a *CHA1* probe that identifies Chr III. The SMD was estimated in the ethidium bromide stained gel (A) by comparing the amount of DNA material in the SMD region to DNA in the small chromosomes that experienced little breakage, “SMD / Chr (I+VI)”. The MMS-induced breakage of the circular Chr III is indicated on the Southern blot (B). Resection, identified by the upward “PFGE-shift” of linear Chr III in (B), is seen in all three strains. The *rad51*- and *rad52*-dependent generation of dimer-size linear chromsome III is also indicated.

**Figure 5:**
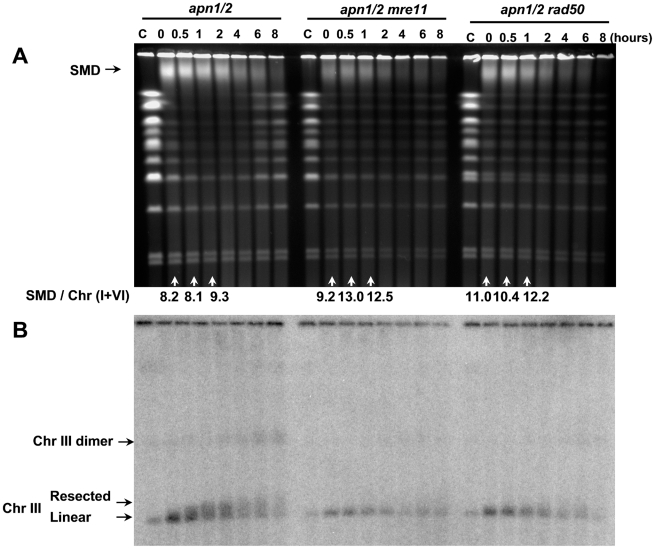
Resection as indicated by PFGE shift requires the MRX complex. Logarithmically growing *apn1/2* and triple mutants *apn1/2 mre11* or *apn1/2 rad50* were arrested in G2/M with nocodazole, treated with MMS (0.1%, 15 min) in PBS and returned to the YPDA+nocodazole medium. Cells were collected at the indicated times and processed for PFGE analysis. Chromosomes were visualized by ethidium bromide staining (A) and by Southern blotting (B) with a *CHA1* probe that identifies Chr III. Resection-related PFGE-shift of linear Chr III and formation of Chr III dimers are indicated (B). The SMD was estimated in the ethidium bromide stained gel (A) by comparing the amount of DNA material in the SMD region to DNA in the small chromosomes that experienced little breakage, “SMD / Chr (I+VI).”

**Figure 6:**
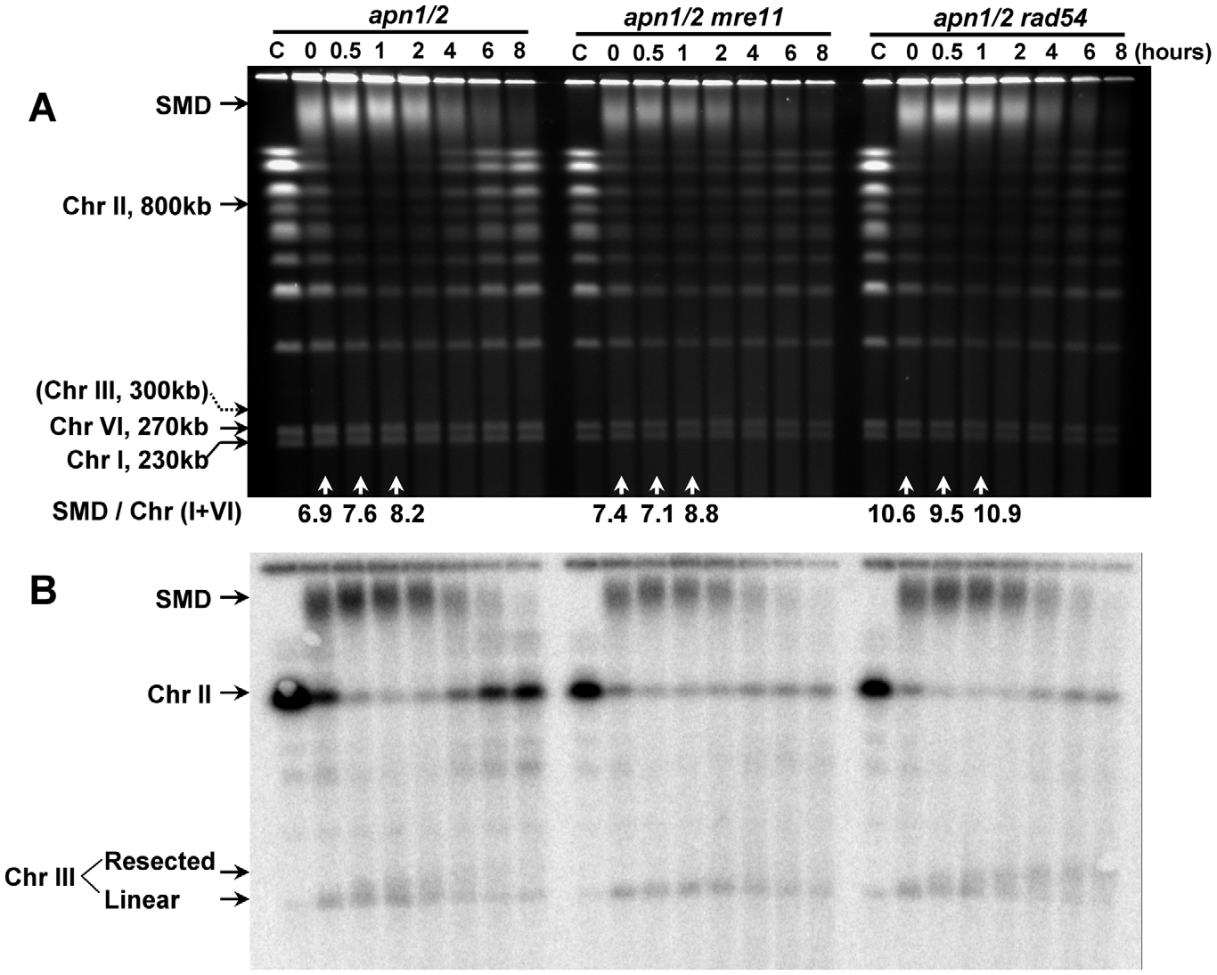
The appearance of SMD is much great for a larger (Chr II) than a smaller chromosome (Chr III). Logarithmically growing *apn1/2* and triple mutants *apn1/2 mre11* or *apn1/2 rad54* were arrested in G2/M with nocodazole, treated with MMS (0.1%, 15 min) in PBS and returned to the YPDA+nocodazole medium. Cells were collected at the indicated times and processed for PFGE analysis. Chromosomes were visualized by ethidium bromide staining (A) and by Southern blotting (B) with a *LEU2* probe that identifies both ChrII and III. Resection-related PFGE-shift of linear Chr III and formation of Chr III dimers are indicated (B). The SMD was estimated in the ethidium bromide stained gel (A) by comparing the amount of DNA material in the SMD region to DNA in the small chromosomes that experienced little breakage, “SMD / Chr (I+VI).”

To further address how DSBs are generated by MMS in the *apn1/2* mutant and their repair in G2/M cells as well as the mechanism of SMD appearance and loss, we first investigated whether HR has a role in these processes. Deletions of key genes involved in HR including *RAD50*, *-51*, *52*, *-54* and *MRE11* were generated in the *apn1/2* background. While HR mutants are MMS sensitive even in an APN^+^ background [33], they do not affect the appearance of MMS-induced chromosomal damage or repair in G1 stationary cells as compared to wild type cells [28] because of efficient BER. The reports of MMS sensitivity of HR mutants are likely due to small number of lesions that remain unrepaired when cells pass into S-phase [34]. Nothing is known about the induction and repair of MMS derived DSBs in G2 cells where there are opportunities for recombinational repair between sister chromatids. Similar to results in G1 cells, there appeared to be little or no induction of derived DSBs when *rad52* APN^+^ cells were treated in the G2/M phase of the cell cycle (Figure S1). However, efficient restitution of full-length chromosomes in the *apn1/2* cells treated with MMS in G2/M does require components of the recombinational repair pathway as shown in Figure 4A (*rad52* and *rad51*), Figure 5A (*mre11* and *rad50*) and Figure 6A (*mre11* and *rad54*). The small amount of chromosome restitution in some of the mutants might be due to some sort of microhomology mediated end-joining given the *RAD51* independence and *RAD52* dependence. Thus, in contrast to the situation in G1 cells, where there are no opportunities for recombination, derived DSBs created in G2 cells by MMS can undergo recombinational repair between sister chromatids (also addressed below using an *mcd1* cohesin mutant).

The gain and loss of SMD in the *apn1/2* mutant appeared to parallel the timing of chromosome breakage and restitution, as shown in Figure 4A. However, SMD formation is not related to recombination since additional deletion of *RAD52*, *RAD51* (Figure 4A), *RAD50*, *MRE11* ((Figure 5A), or *RAD54* (Figure 6A) did not significantly alter the appearance of SMD. To compare the levels of SMD in response to MMS and between strains, we determined the ratios between amount of material in the SMD region to DNA in the small chromosomes that experienced little breakage. As shown at the bottom of panel A in each figure (“SMD/Chr I+VI”), the ratios were similar between the *apn1/2* and the various triple mutants over the first hour of treatment. In all cases, there was considerable reduction in SMD by 4 hours. The decreased amount of SMD in *rad52* or *mre11* (in Figure 4A, Figure 5A) could be due to delays in S-phase arising from repair defects which would lead to an overall reduction in DNA entering into the gel since the mutants have a somewhat reduced growth rate. We also investigated the 5′ to 3′ exonuclease I (*EXO1*) since it can resect at random DSBs and appears to enlarge single-strand gaps during nucleotide excision repair [35] that could lead to reduced chromosomal DNA mobility during PFGE. As shown in the Figure S2, exonuclease 1 does not influence either the repair of the MMS induced DSBs or the appearance of SMD.

The role of HR in repair of the MMS-derived DSBs and the lack of contribution to the appearance and loss of SMD is investigated further in Figure 6B. Using a *LEU2* probe for Chr II+III, it is clear that nearly all the DNA of Chr II appears as SMD within 30 minutes after treatment. (This probe, which identifies Chr II, also hybridizes with circular and broken Chr III molecules, as described in Ma *et al.* [28] and is discussed below.) The amount of SMD started to decrease at 2 hours after MMS exposure and a significant amount of SMD is lost by 4 hours, where at the same time there is a reappearance of full size Chr II. However, there is substantial restitution of Chr II starting at 4 hours in the *apn1/2* mutant, but not in the *mre11* and *rad54* derivatives. Thus, while the appearance and loss of SMD are not influenced by HR, based on the Southern blotting results with the *apn1/2 mre11* and *apn1/2 rad54*, the reappearance of full-size Chr II requires HR.

### MMS–derived DSBs are resected and can generate recombinant molecules

While a role for recombinational repair of MMS-associated DSBs has been proposed for S-phase cells and demonstrated above for G2/M cells, there has been no direct demonstration of DSB processing or generation of recombinants. This is due in part to the difficulties of characterizing events associated with random DSBs (discussed in [29]). In addition, opportunities to examine MMS-induced events in S-phase cells using PFGE are limited because of the structures created in the replicating DNA which results in most of the DNA being retained in the starting wells [36].

Recently, we described an assay involving PFGE and circular chromosomes for characterizing resection and recombination at random DSBs [29]. Since a single DSB in a circular chromosome results in a unit length linear molecule, the direct or derived induction of random DSBs can be followed by the appearance of the corresponding band with PFGE (as described in [28], [29], [37]). Importantly, resection at the DSB ends leading to the generation of single-strand tails could be detected by reduced mobility of the unit length molecules (*i.e.*, “PFGE-shift”). Previously, we showed that MMS treatment of *apn1/2* stationary G1 cells led to the linearization of the circular Chr III. However these “DSBs” could have arisen from molecules with closely spaced-SSBs during preparation of chromosomal material for PFGE analysis. The resection in the G2 cells, as well as subsequent repair, establishes that MMS-induced DSBs actually occur *in vivo* in G2 cells.

Similar to the results with the G1 stationary cells, MMS treatment of *apn1/2* cells resulted in the rapid appearance of linear Chr III molecules in Southern blots using a probe specific to this chromosome (Figure 4B and Figure 5B). However, unlike observations with *apn1/2* cells treated in G1 [28], the linearized molecules from the G2/M cells exhibited the PFGE-shift similar to that found previously for direct DSBs induced by IR [29], suggesting that the MMS derived DSBs are subject to resection. The PFGE shift which appeared by 1 hour after treatment was also found in the *apn1/2 rad52* and *rad51* triple mutants (Figure 4B). The bulk of PFGE-shift required MRX (Mre11, Rad50, Xrs2) since there was no apparent resection with the *apn1/2 rad50* or *mre11* mutants (Figure 5 and Figure 6). The PFGE profiles of DSBs induced by MMS in the triple mutants of *apn1/2* combined with deletions of the recombinational repair genes were similar to patterns found for *rad52*, *rad51*, *rad50* and *mre11* single mutants exposed to IR [29]. Thus, the MMS derived DSBs are subject to resection; MRX plays an important role, presumably through initiation of 5′ to 3′ resection. The processing of DSBs induced by IR appears different from that found for MMS-derived DSBs in that the PFGE shifted band is smeared as compared to a narrow shifted band for IR damage (*e.g.*, the *rad52* mutant; [29]). The smearing of the band after MMS treatment might be due to the formation of single-stranded tails with variable lengths or the timing of DSB formation and resection (Figure 4B).

Similar to our findings with radiation-induced direct DSBs [29], the MMS treatment of *apn1*, *2* cells leads to the appearance of linear Chr III molecules at 2 to 4 hours post-treatment that are twice the size of the broken Chr III (Southern blots in Figure 4B and Figure 5B). Based on our previous studies [29], these dimers are likely the product of recombination between full size Chr III sister chromatids (discussed in [29]). The role of recombination is supported by the observed dependence on Rad51 and Rad52 (Figure 4A). Overall these results provide the first direct physical evidence of i) MMS lesions being processed to DSBs in G2 cells, ii) resection of the ends, and iii) MMS generation of recombinant molecules.

### Role of *NTG1*, *NTG2*, and *OGG1* in formation of DSBs and SMD

**Figure 7:**
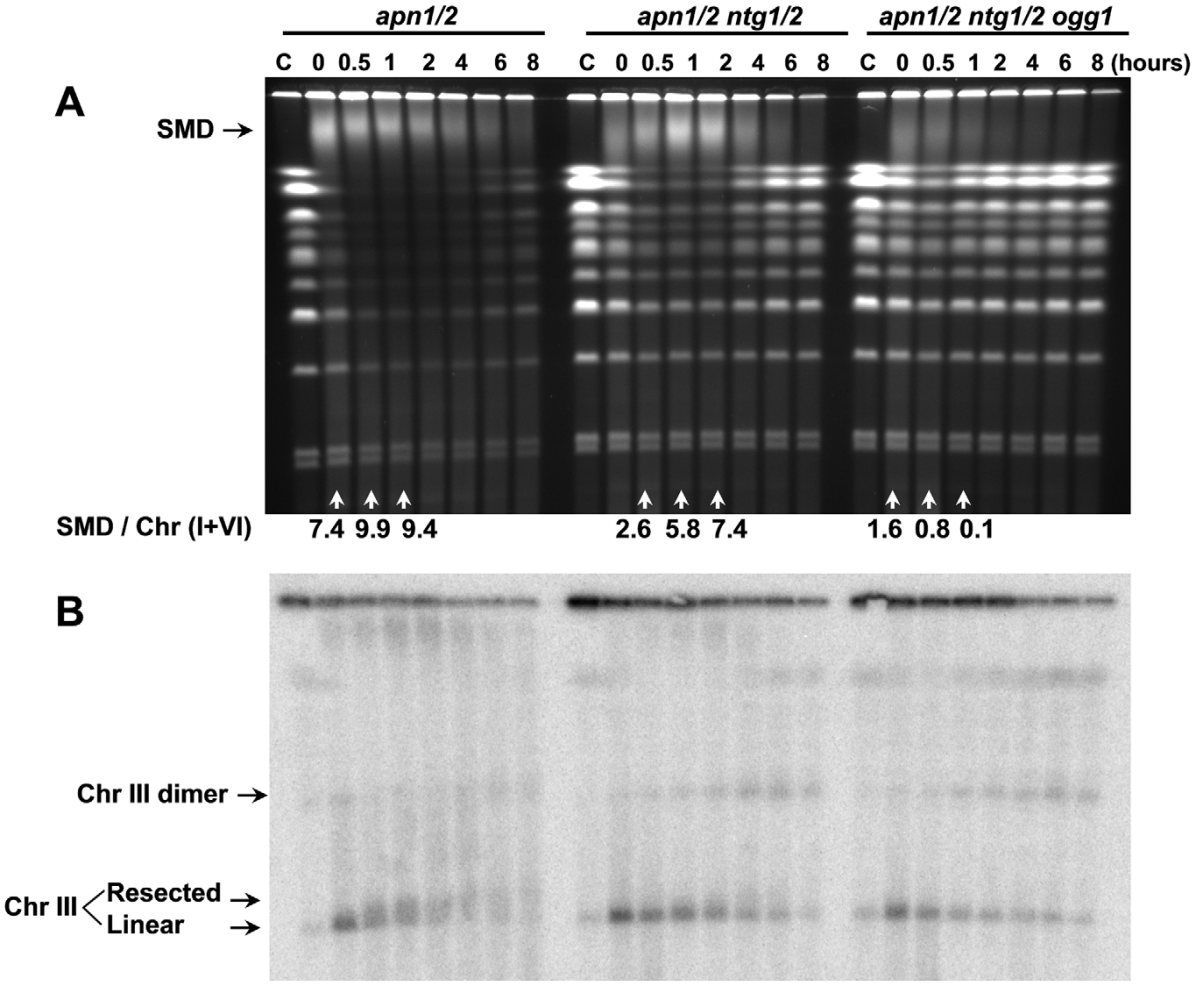
Deletion of *NTG1*, *NTG2*, and *OGG1* inhibited the formation of DSB and SMD. Logarithmically growing *apn1/2*, *apn1/2 ntg1/2* and *apn1/2 ntg1/2 ogg1* were arrested in G2/M with nocodazole, treated with MMS (0.1%, 15 min) in PBS and returned to the YPDA+nocodazole medium. Cells were collected at the indicated times and processed for PFGE analysis (A). Southern blot analysis of the gel used the Chr III specific probe *CHA1* (B). Resection-related PFGE-shift of linear Chr III and formation of Chr III dimers are indicated (B). The SMD was estimated in the ethidium bromide stained gel (A) by comparing the amount of DNA material in the SMD region to DNA in the small chromosomes that experienced little breakage, “SMD / Chr (I+VI).”

In the absence of the Apn1 and Apn 2 endonucleases, AP sites can be nicked at the 3′ side by the bifunctional DNA N-glycosylases/AP lyases Ntg1 and Ntg2 that convert AP sites into 3′-blocked SSBs [13]. Additionally, the bifunctional 8-oxyguanine glycosylase Ogg1 can nick an AP site that is opposite a cytosine [38]. Mutants of these genes were created in the *apn1/2* background to identify possible contributors to the appearance of DSBs and SMD. As shown in Figure 7, the *apn1/2 ntg1/2* quadruple mutant also exhibited appearance and loss of SMD. However, there was less chromosome breakage and nearly full chromosome restitution by 4 hours, as compared to the incomplete chromosome restitution in the *apn1/2*, even after 8 hours. The additional *ntg1/2* mutations increased the time required for maximal appearance of SMD and there is less material lost in the full-size chromosome bands, consistent with a decreased likelihood of single-strand break generation. The increased resistance of *apn1/2 ntg1/2* compared to *apn1/2* cells suggests that DSBs rather than SMD are the major contributor to loss of survival with or without arrest in G2 following MMS treatment (Figure 2; the dose-modifying factor is ∼3). The combination of *apn1/2 ntg1/2* does not totally block formation of DSBs. While there appears to be less processing of ends, recombinants can still be formed based on the formation of Chr III dimers (Figure 7B). A similar finding of dimer generation, independent of resection, was reported for MRX mutants following IR [29].

Further deletion of *OGG1* (*i.e.*, *apn1/2 ntg1/2 ogg1*) greatly reduced the appearance of SMD formation and decreased DSB induction (Figure 7). Survival was also improved compared to the *apn1/2 ntg1/2* mutant (Figure 2). Thus, SSBs generated at AP sites are a likely source of SMD consistent with the above findings with the *apn1/2 mag1* mutants as well as the effect of MX (Figure 3) where AP sites are prevented or blocked. Furthermore, these results demonstrate a role for *OGG1* in the general processing of methylated base damage or imply that MMS causes additional types of damage that are substrates for Ogg1. The reduced amount of overall chromosome breakage observed with the ethidium bromide stained gel (Figure 7A) is expected if there are less SSBs to generate derived DSBs. Surprisingly, some linearized Chr III molecules and dimers were generated, based on Southern blotting with a probe specific to Chr III, even though there is no resection. Possibly they were formed through additional mechanisms for processing abasic sites and/or *in vitro* during PFGE of DNAs with opposed SSBs that are sufficiently close.

### A defect in sister chromatid cohesion reduces chromosome restitution but not SMD

While the results with the various RAD mutants demonstrate that neither the appearance nor the disappearance of SMD is dependent on HR, there is still the possibility of SMD arising through unknown interactions with sister chromatids. To address this, a temperature-sensitive cohesin mutant *mcd1-1* was generated in both a WT and *apn1/2* background. Cohesin is required to hold sister chromatids together and is essential for efficient repair of radiation induced DSBs [39]–[41]. The *mcd1-1* single mutant grows well at permissive temperature of 23°C but is not viable at 37°C [42], consistent with our observation of no growth of the *apn1/2 mcd1-1* triple mutant at the elevated temperature. In preliminary experiments we found that *apn1/2* cells exhibited less repair of MMS lesions at 37°C. This may be due to more closely-opposed lesions being converted into DSBs, since methylated bases are heat-labile. Therefore, cells were incubated at 37°C for 3 hours to inactivate the temperature-sensitive cohesin; during this period cells were arrested in G2/M by nocodazole. Cells were then shifted to the semi-permissive temperature of 30°C for MMS treatment as well as post-MMS incubation in YPDA with nocodazole.

**Figure 8:**
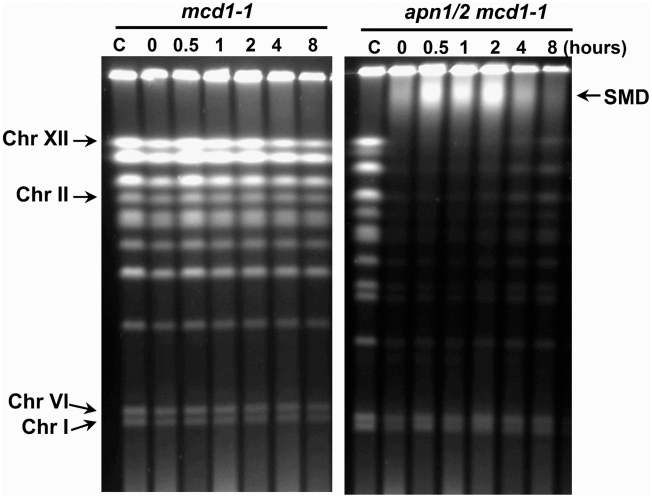
The appearance of SMD is not affected by a defect in cohesion. The temperature-sensitive *mcd1-1* and *apn1/2 mcd1-1* cells were grown overnight at permissive temperature (23°C) in YPDA media. The logarithmically growing cells were shifted to non-permissive temperature (37°C), nocodazole was added and cells were incubated another 3 hours to arrest them at G2/M. Cells were then treated with MMS (0.1%, 15 min) in PBS at semi-permissive temperature (30°C) followed by incubation in YPDA with nocodazole. Cells were collected at the indicated times and processed for PFGE analysis.

As shown in Figure 8, MMS did not lead to the appearance of DSBs or loss of chromosomes in the *mcd1-1* single mutant, similar to results with wild type cells (Figure 1). As expected, derived DSBs were detected in the *apn1/2 mcd1-1* triple mutant. However, unlike observations with the *apn1/2* mutant (Figure 1) there was much less overall restitution of chromosomes at 4 and 8 hours, more similar to the *apn1/2 rad51* mutant (Figure 4). Yet, the formation and disappearance of SMD was not affected. Most chromosome bands, especially the larger chromosomes, were lost from the PFGE gels in the *apn1/2 mcd1-1* triple mutant but not in the *mcd1-1* single mutant (Figure 8), which is comparable to results with other HR mutants. These findings suggest that cohesin is not required during SMD formation and that SMD is not a consequence of sister chromatid interaction.

### Structures generating SMD are infrequent and are sensitive to T7 endonuclease I

As described above, the generation of SSBs at abasic sites is required for the generation of SMD. However, the SMD molecules do not rely on sister chromatid interactions, recombination or DSB ends, based on a lack of SMD at later times in the HR mutants (Figure 4, Figure 5, and Figure 6). Examination of several of the ethidium bromide gels, reveal that SMD is dependent on chromosome size (see, for example, Figure 1 and Figure 4A). In several of the experiments there is little disappearance of the smallest chromosomes (I and VI). This is confirmed in Figure 6 where Chr II (800 kb) and linearized Chr III can be detected with a common probe. Nearly all the larger Chr II molecules appear in SMD, while there is little change in the amount of the smaller Chr III. This is further substantiated in Figure 4 and Figure 5, where only Chr III is probed and there is little, if any SMD. With a decrease in SSBs, there is less SMD with only the large chromosomes being affected (Figure 7). Thus, the SSB related lesion(s) or combination of lesions leading to SMD appear to be less than an average of 1 per few hundred kb.

**Figure 9:**
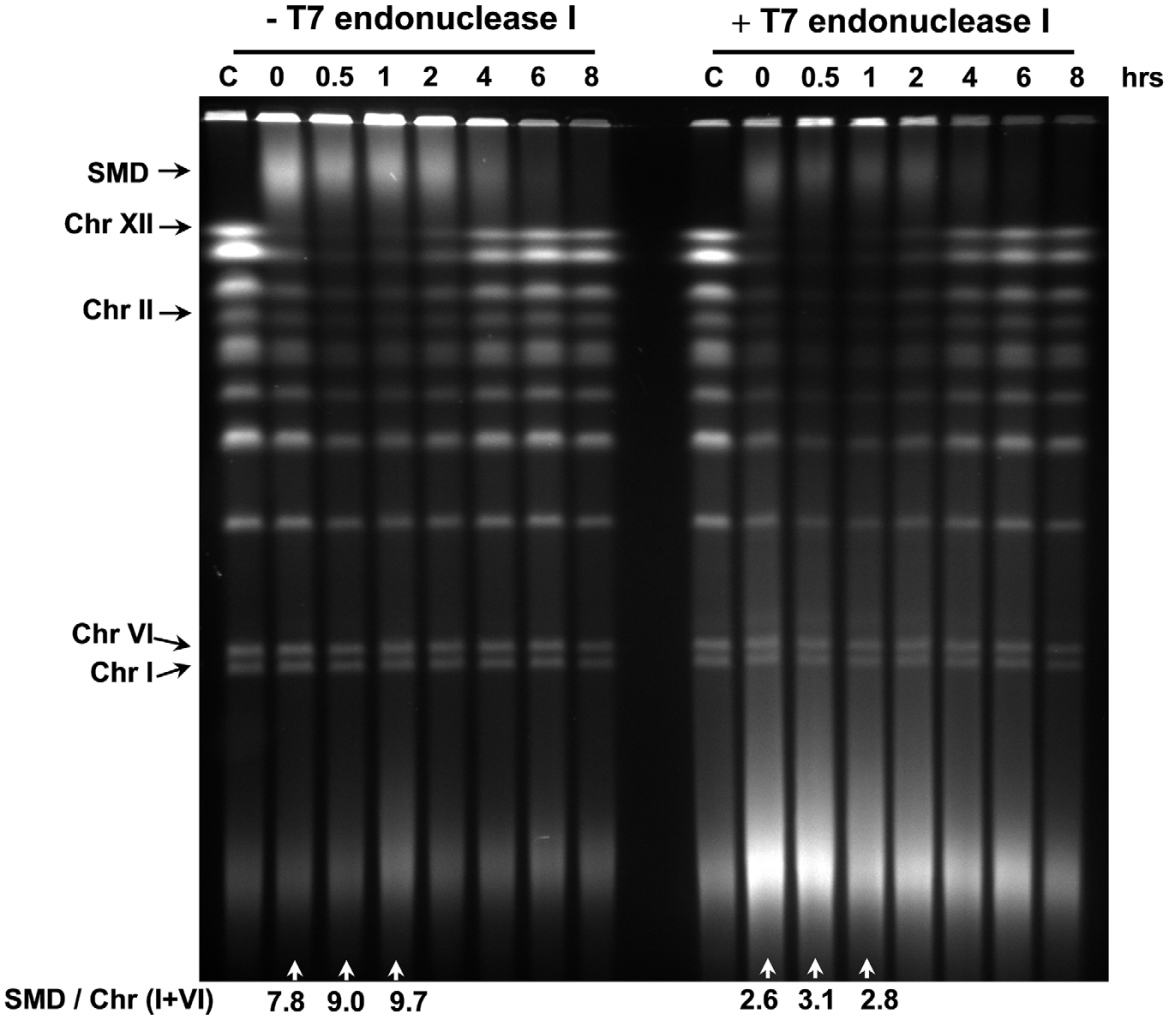
SMD is sensitive to T7 endonuclease I. Logarithmically growing *apn1/2* cells were arrested in G2/M with nocodazole, treated with MMS (0.1%, 15 min) in PBS and returned to the YPDA+nocodazole medium. Cells were collected at the indicated times and processed for DNA plug preparation. The plugs were then incubated with T7 endonuclease I as described in the Materials and Methods or in buffer only (mock-treated). The SMD was estimated in the ethidium bromide stained gel by comparing the amount of DNA material in the SMD region to DNA in the small chromosomes that experienced little breakage, “SMD / Chr (I+VI)”.

Given the retardation of much of the chromosomal DNA in PFGE, we investigated various enzymes that recognize structural changes in DNA to help discern the nature of SMD. (We also considered proteins bound to DNA that could give rise to SMD; however, we found that extending the proteinase K treatment beyond that normally used in preparation of plugs for PFGE did not change the SMD as noted in “Material and Methods”.) Mung bean nuclease had been used to demonstrate resected DNA at radiation induced DSB ends [29]. However, for the DNA obtained after MMS treatment, there was general degradation by this nuclease of the chromosomal DNA treated in plugs before PFGE. This extensive digestion is likely due to this nuclease acting at SSBs and possibly gap-like structures. We also investigated bacteriophage T7 endonuclease I because of its ability to recognize and cleave at a variety of structures including DNA mismatches, nicks as well as branch molecules such as Holliday type junctions [43]–[45]. As shown in Figure 9, endonuclease I treatment of the chromosomal DNAs following MMS treatment of *apn1/2* cells eliminated much of the SMD leading to the appearance of small DNA fragments (∼50 to 100 kb). This enzyme specifically acted on SMD since there was little effect on the chromosomal DNA from untreated cells or the chromosomal DNA following 4 hour to 8 hour of repair. The cutting of SMD into small molecules by T7 endonuclease I (*i.e.*, smaller than the Chr I and Chr VI which exhibit little SMD) suggests the presence of few endonuclease responsive substrate structures in the smaller chromosomes. Interestingly, the use of T7 endonuclease I to remove the SMD enabled us to establish further that repair of derived DSBs does occur between 2 and 4 hours (as previously suggested in Figure 4, Figure 5, Figure 6). The SMD structures sensitive to T7 endonuclease I are unlikely to be due to recombinational intermediates because SMD was observed in the various HR mutants as described above. While they could be related to branched molecules produced from persisting nicks, other possibilities exist given the various types of structures susceptible to this endonuclease.

## Discussion

BER is critical for dealing with a variety of single strand lesions. Many enzymes in this pathway are conserved from microorganisms to humans and serve as antimutators, especially in terms of tumor suppression and preventing hereditary neurodegenerative disease [46], [47]. Aberrant BER processes might result in the eventual appearance of DSBs, which are a major source of genome instability. MMS-induced lesions are considered a source of DSBs as a result of collapsed replication forks at the lesions or processed intermediates. Based on genetic evidence, these replication-associated DSBs have been considered to be repaired by HR mechanisms.

Previously, we showed that MMS-induced single-strand damage in G1 arrested cells had the potential for generating derived DSBs and highlighted the role that Rad27 and Pol32 play in preventing such breaks [20]. We had concluded that closely-spaced opposing lesions could be a source of the derived DSBs and that well-coordinated BER assures prevention of these downstream DSBs. The present study using G2/M cells is the first to characterize directly the generation, processing and repair of derived DSBs following treatment by an alkylating agent. While two closely-opposed SSBs with 5′-blocked termini could be “moved closer” to form a DSB during repair-associated DNA synthesis and strand displacement [17], [18], [19], [20], [21], this is not expected to be the case for SSBs with 3′-blocked termini since they would not support DNA synthesis directly. The present results are consistent with derived DSBs resulting from generation of SSBs at closely opposed lesions. It is also possible that derived DSBs could arise through processing of more distant SSBs with 3′-blocked ends in cells lacking AP endonucleases, in essence the breaks are “moved closer,” as discussed below. Furthermore, we have described a novel repair intermediate, SMD, which can be generated if abasic sites are nicked by AP lyases.

While the *APE1* gene, which codes for the major mammalian AP endonuclease (the APE1 homologue APE2 has only weak endonuclease activity, and its role in human BER is not clear), is essential for human cell survival and results in embryonic lethality when knocked out in mouse [48]–[51], yeast can survive the deletion of both AP endonucleases with almost no growth defect. It is, therefore, possible to study alternative mechanisms/pathways that deal with AP sites and 3′-blocked SSBs *in vivo* and their role in generating DSBs. Earlier studies had demonstrated that MMS does not cause DSBs directly [28], [30]. With an assay that can specifically monitor the processing of closely-opposed single strand lesions, our previous study showed that PFGE-detected DSBs were accumulated in G1 *apn1/2* haploid yeast after MMS damage. However, since closely-opposed SSBs might lead to chromosome DNA breakage during *in vitro* handling, the extent to which the PFGE-detectable DSBs were actually formed *in vivo* remained a question. Here, we confirm that DSBs do appear after MMS treatment of G2 cells lacking AP endonucleases, as demonstrated by i) resection, ii) a requirement for HR components to reconstitute chromosomes, and by iii) the formation of Chr III dimers. While resection is generally considered essential for DSB repair mechanisms [52], [53], we have demonstrated that it also occurs at the MMS-derived DSBs and like radiation-induced DSBs they are subject to MRX control. As in the case of randomly generated radiation-induced DSBs [29], we aimed to determine other factors affecting resection at the MMS-derived DSBs, especially factors that may lead to increased resection. We have recently shown that UV as well as MMS damage to single-strand DNA formed at site-specific DSBs cause high level of mutagenesis [4], [5]. Increasing resection at MMS derived breaks could further enhance its mutagenic potential.

The current results further confirm that DSBs can be derived from AP sites arising during BER, since the appearance of DSBs could be blocked either at the step in which methylated bases are removed or if cleavage of AP sites is prevented by MX (Figure 3). It is clear that DSBs were generated by the bifunctional glycosylases because deletion of *NTG1*, *NTG2* and *OGG1* along with *APN1* and *APN2* blocked the formation of DSBs as well as resection. The targets of these enzymes are limited to AP sites instead of methylated bases based on efficient DSB inhibition following deletion of *MAG1* (Figure 3). Though *OGG1* is known to deal primarily with oxidative damage [14], [54], we have shown that this bifunctional glycosylase provides a backup for cleavage at AP sites following induction of MMS damage since derived DSBs that appeared in the *apn1/2 ntg1/2* mutant were prevented by a further *ogg1* mutation (Figure 7). This is the first direct demonstration for the Ogg1 glycosylase dealing with lesions other than oxidative damage *in vivo*, suggesting a potentially more general role for this gene in repair. Considering that the predominant lesions induced by MMS are N7-methylguanine and N3-methyladenine [55], the function of Ogg1 in the development of DSBs and SMD is likely due to its action on AP sites derived from N7-methylguanine. There was still a small amount of DSBs after removal of all the bifunctional glycosylases/lyases (Figure 7) which might be due to NER or some other enzymes. It was shown that DNA Topoisomerase I (Top1) forms DNA-protein adducts with nicked and gapped DNA structures [56], [57]. Possibly the AP sites could also be processed by yeast topoisomerases to generate DSBs. This might explain the small amount of SMD presented in *apn1/2 ntg1/2 ogg1* mutants (Figure 7).

**Figure 10:**
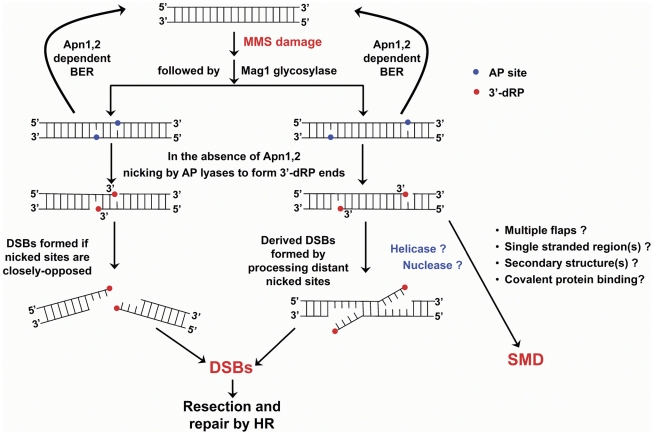
Scheme for the appearance of DSBs and SMD. Methylated bases are first removed by Mag1 glycosylase to form AP sites. In *apn1/2* mutants AP sites are nicked by AP lyases (Ntg1, Ntg2 and Ogg1). If two nicks are close enough, they would result in a derived DSB and undergo resection and HR mediated repair. When the two nicks are not closely-spaced, a derived-DSB might still be formed during attempted removal of the α,β-unsaturated aldehyde (3′-dRP) possibly by helicases that generate a single-stranded 3′-flap, and nucleases or other proteins that remove the blocked group and restore a ligatable 3′-end. Inefficient removal of multiple flaps, secondary structures from single stranded region or flaps as well as stably bound proteins might result in molecules with retarded mobility (*i.e.*, SMD).

As summarized in Figure 10, the generation of derived DSBs would require that opposed AP sites either be sufficiently close (left side of figure) so that DSBs are created directly *in vivo* or there is a nick-processing mechanism that “moves” the relatively distant opposing-nicks closer (central part of figure) to form a DSB. Considering that MMS is an SN2 type of alkylating agent that methylates DNA bases in a random manner with a limited ability to produce closely-spaced lesions under the conditions used in this study (in contrast to ionizing radiation [58]), many of the MMS-derived DSBs might be generated from distant single-strand breaks during processing/repair of the end-blocking groups as also suggested from our previous study with *rad27* and *pol32* mutants [20]. Since AP lyases generate blocked 3′-ends (3′-dRP) while repair of either SSBs or DSBs requires an unblocked 3′-OH end for repair synthesis or ligation, we suggest that both the formation of DSBs and SMD are related to the processing of 3′-blocking groups. Either or both might be generated through development of 3′-flaps, possibly by helicases or nucleases. For example, opposing SSBs could be “moved” together to form a DSB if 3′-flaps are generated toward each other. Possibly it is the generation of multiple flaps that leads to the reduced mobility of large DNAs on PFGE, and the SMD molecules; however, the reduction in mobility is not as great as observed with replicating chromosomes, which remain in the well during PFGE. Although exonuclease 1 generated gaps at UV-damage sites can lead to reduced mobility, they are unlikely to be the source of SMD in the present experiments, While we have shown that the DSBs and SMD arise from a common BER intermediate, their subsequent appearance and disappearance are genetically separable. Importantly, we have established that SMD does not involve the HR pathway. The derived DSBs are subject to processing by MRX and the subsequent DSB repair as well as the appearance of dimer recombination products requires HR.

Regardless, there are limitations on the appearance of SMD. The loss of chromosomal DNA along with the appearance of the wide band of SMD following MMS treatment of *apn1/2* cells is dependent on the size of the chromosome. SMD was substantially greater for larger chromosomes than smaller ones (Figure 6). This is clearly shown in a Southern blot comparison of linearized Chr III with Chr II and in comparisons of the 230 kb Chr I and 270 kb VI with the larger chromosomes where there was little if any loss of the smaller chromosome bands (Figure 6). Based on our previous results [28], we anticipate ∼0.4 SSBs/kb which would lead to considerable damage in even the smallest chromosomes (∼100 SSBs/Chr I). Thus, while SMD requires the generation of SSBs, other factors determine its appearance. Possibly, the appearance of SMD depends simply on the likelihood of producing some minimum amount of lesions or certain types of structures (*i.e.*, sensitive to T7-endonuclease) that are stable *in vitro*.

The requirement for generation of a 3′-flap to remove 3′-blocked termini had been proposed previously to explain the synthetic lethality between *apn1/apn2* and *rad1* or *rad10* [25], [59]. Although *in vitro* studies demonstrated that a 3′-flap can be removed by Rad1/Rad10 proteins [60], direct evidence for flap removal *in vivo* has been lacking. The observation of SMD in our current study fits well with this hypothesis though the actual mechanism for its formation and release might be more complex than previously proposed. It is interesting that while we have eliminated SMD as a recombination product, it is sensitive to the T7 endonuclease I which can cleave structures that might arise during recombination as well as branched molecules containing single strand regions (possibly as a result of 3′-flap formation as proposed in Figure 10).

In conclusion, our study identifies new mechanisms for processing abasic sites and provides the first direct demonstration in nonreplicating G2/M cells of MMS-derived DSBs and that the DSBs are subject to recombinational repair. In addition, we identify and characterize the generation of SMD. While not previously described, possibly because of the techniques used to assess DNA damage and repair, SMD might be a general repair intermediate for various types of DNA damage, a view that we are currently pursuing. Interestingly, there has been an indication, though not directly addressed, of SMD-like material in exonuclease 1 defective yeast cells during excision repair of UV damage [35]. The combination of genetics and systems for detection of novel structures has provided a unique opportunity to address processed events at intermediates in repair of DNA lesions. While the derivation and repair of derived DSBs has been addressed as well as the generation of SMD, it will be interesting to determine the specific nature of the actual DNA changes that lead to SMD and the eventual resolution including the genetic controls. To our knowledge, this is the first report of a novel branched repair intermediate being generated during the processing of 3′-blocked termini. These findings are expected to expand our understanding of mechanism for repair of 3′-blocked ends as well as their impact on genome stability.

## Materials and Methods

### Yeast strains

All strains are haploid derivatives of two isogenic haploid yeast strains MWJ49 and MWJ50 (*MAT*α *leu2-3,112 ade5-1 his7-2 ura3D trp1-289*) which contain a circularized chromosome III and has the construct *lys2*::Alu-DIR-*LEU2*-*lys2D5′* on Chr II [28]. The construction of strains with circular Chr III was described in [28]. Deletion strains of *apn1*, *apn2*, *rad50*, *rad51*, *rad52*, *mre11*, *mag1*, *ntg1*, *ntg2*, *ogg1* and derived multiple mutants were created by replacement of the relevant open reading frame with selectable markers by PCR [61]. Temperature-sensitive mutants of *mcd1-1* in wild type or *apn1/2* background were generated using plasmid pVG257 [41]. Experiments were done at 30°C, unless specifically stated at a different temperatue.

### Cell-cycle arrest at G2/M and MMS treatment

The generation of G2 arrested cells was described in [62]. Briefly, logarithmically growing cells in YPDA medium (1% yeast extract, 2% Bacto-Peptone, 2% dextrose, 60 mg/ml adenine sulfate) were incubated with nocodazole at a final concentration of 15 µg/mL. After 3 hours, most cells are arrested in G2/M as determined microscopically by the presence of large budded cells and verification using flow cytometry. Cells were then harvested by centrifugation, washed and resuspended in phosphate-buffered saline (PBS, 10 mM phosphate, 0.138 M NaCl; 0.0027 M KCl, pH 7.4). MMS treatment was performed as described in [28] with modification. Cells in PBS were incubated with 11.8 mM (0.1%) MMS for 15 or 30 min at 30°C with vigorous shaking, and then neutralized by mixing 1∶1 (v/v) ratio with 10% Na_2_S_2_O_3_. After washing with dH_2_0, a portion of the MMS-treated cells was immediately resuspended in ice-cold cell suspension buffer (10 mM Tris (pH 8.0), 100 mM EDTA) to prepare DNA-agarose plug for pulsed-field gel electrophoresis (PFGE) as described below. Other portions of the MMS-treated and control cells were resuspended in YPDA media containing nocodazole and incubated at 30°C with constant shaking. Cells were collected up to 16 hours after MMS treatment, centrifuged, wash with dH_2_0 and resuspended in cell suspension buffer for PFGE DNA-agarose plug preparation.

### Methoxyamine (MX) treatment to inhibit incision at AP sites

Nocodazole-arrested G2 cells were first incubated with MX (final concentration 100 mM) in YPDA for 15–30 min to first allow MX diffuse into cells. Then MMS treatment and post-treatment incubation were as described above with MX (final concentration 100 mM) present during the whole procedure. Cells were then collected at various times for plug preparation and PFGE analysis.

### PFGE analysis of chromosomal DNA damage and repair

Detection of DSBs and repair intermediates (such as resected DNA molecule) were based on PFGE analysis as described [28]. PFGE was performed using a Bio-Rad CHEF-Mapper XA system (Bio-Rad, Hercules, CA). Preparation of agarose-embedded DNA (DNA plug) was described in [28]. Briefly, control and MMS-treated cells collected at different times following MMS treatment were embedded in 0.6% agarose with 1 mg/ml Zymolyase (100 U/mg, MP Biochemicals, Solon, OH). The plug was incubated for 1 h at 30°C in a “spheroplasting” solution (1 M sorbitol, 20 mM EDTA, 10 mM Tris pH 7.5) to remove the cell wall. This was followed by digestion with proteinase K (10 mM Tris, pH 8.0, 100 mM EDTA, 1.0% N-lauroylsarcosine, 0.2% sodium deoxycholate, 1 mg/ml proteinase K) for 24 hours at 30°C. Increasing the time of proteinase treatment did not influence the DNA mobility characteristics on PFGE. The parameters for CHEF gel separation of yeast chromosomes in a 1% agarose gel were 6 V/cm for 24 hours with a 10–90 sec switch time ramp and 120° switch angle (running buffer at the 14°C). Subsequently, the DNA was analyzed by Southern blotting as described in [28]. Hybridization was carried out with a probe for the *CHAI* gene to detect specifically Chr III material or a probe to the *LEU2* gene that marked both Chr III and Chr II. Autoradiographs were digitized and densitometric analysis was performed using Kodak MI software (version 5.0).

### Resolution of the slow moving DNA (SMD) intermediate with T7 endonuclease I

DNA was digested in agarose plugs with T7 endonuclease I (New England Biolabs, Beverly, MA). A 50 µl plug slice was equilibrated 3 times for 20 minutes at room temperature in 150 µl of TE (10 mM Tris, pH 7.4, 1 mM EDTA), followed by 30 minute incubation at room temperature with 30 units T7 endonuclease in 150 µL reaction buffer, and stopped by washing 3 times with ice-cold Tris-EDTA (10 mM Tris, 50 mM EDTA, pH 8.0). PFGE analysis was performed as described above.

## Supporting Information

Figure S1 MMS does not generate derived DSBs in WT or *rad52Δ* strains that are *APN^+^*. Logarithmically growing *WT* and *rad52Δ* cells were arrested in G2/M with nocodazole, treated with MMS (0.1%, 20 min) in PBS and returned to the YPDA+nocodazole medium. Cells were collected at the indicated times and processed for PFGE analysis. Chromosomes were visualized by ethidium bromide staining (A) and by Southern blotting (B) with a *LEU2* probe that identifies both ChrII and ChrIII.
(TIF)

Figure S2 Deletion of *EXO1* does not affect the appearance or disappearance of SMD. Logarithmically growing *apn1/2* and *apn1/2 exo1* cells were arrested in G2/M with nocodazole, treated with MMS (0.1%, 20 min) in PBS and returned to the YPDA+nocodazole medium and incubated for up to 8 hours. Cells were collected at the indicated times and processed for PFGE analysis. Chromosomes were visualized by ethidium bromide staining. The slow moving DNA (SMD) was detected as a wide band of DNA as indicated.
(TIF)

Figure S3 Spontaneous depurination of methylated bases in the absence of *MAG1* contributes little to MMS-induced derived DSBs. Logarithmically growing *apn1/2 mag1* cells in YPDA were arrested at G2/M by nocodazole, treated with MMS (0.1%, 15 min) in PBS, returned to YPDA+nocodazole and incubated further. Cells were collected at the indicated times and processed for PFGE and Southern blot analysis with the Chr III specific probe *CHA1*. The induction of DSBs was determined by the appearance of the linear Chr III band.
(TIF)
