# Supplementary figures and images for: Alkylation Base Damage Is Converted into Repairable Double-Strand Breaks and Complex Intermediates in G2 Cells Lacking AP Endonuclease
Source: PLoS Genet. 2011 Apr 28;7(4):e1002059. doi: 10.1371/journal.pgen.1002059 (PMC3084215; doi:10.1371/journal.pgen.1002059)

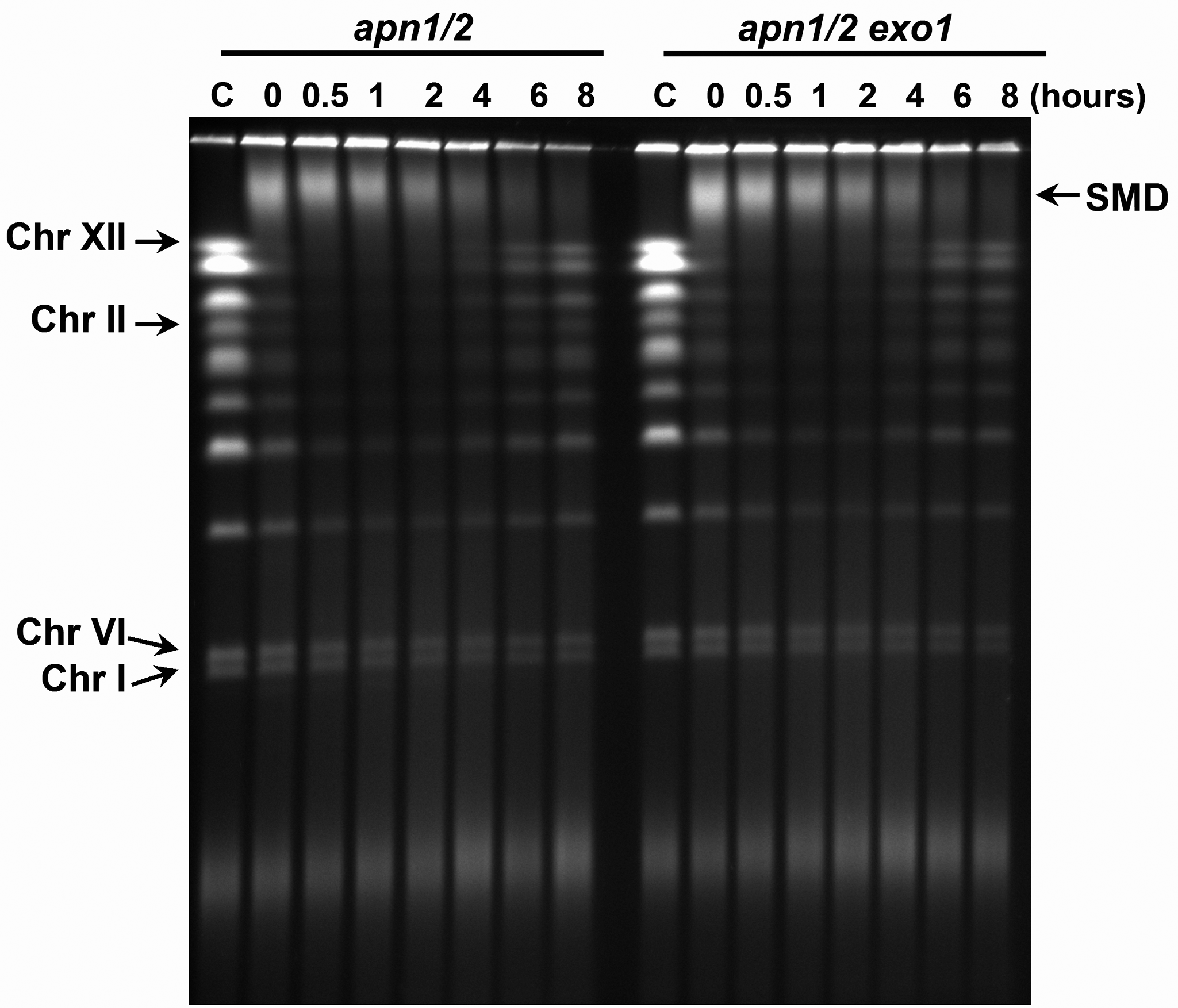

Supplement: Figure S2 — Deletion of EXO1 does not affect the appearance or disappearance of SMD. Logarithmically growing apn1/2 and apn1/2 exo1 cells were arrested in G2/M with nocodazole, treated with MMS (0.1%, 20 min) in PBS and returned to the YPDA+nocodazole medium and incubated for up to 8 hours. Cells were collected at the indicated times and processed for PFGE analysis. Chromosomes were visualized by ethidium bromide staining. The slow moving DNA (SMD) was detected as a wide band of DNA as indicated. (TIF) [file pgen.1002059.s002.tif]

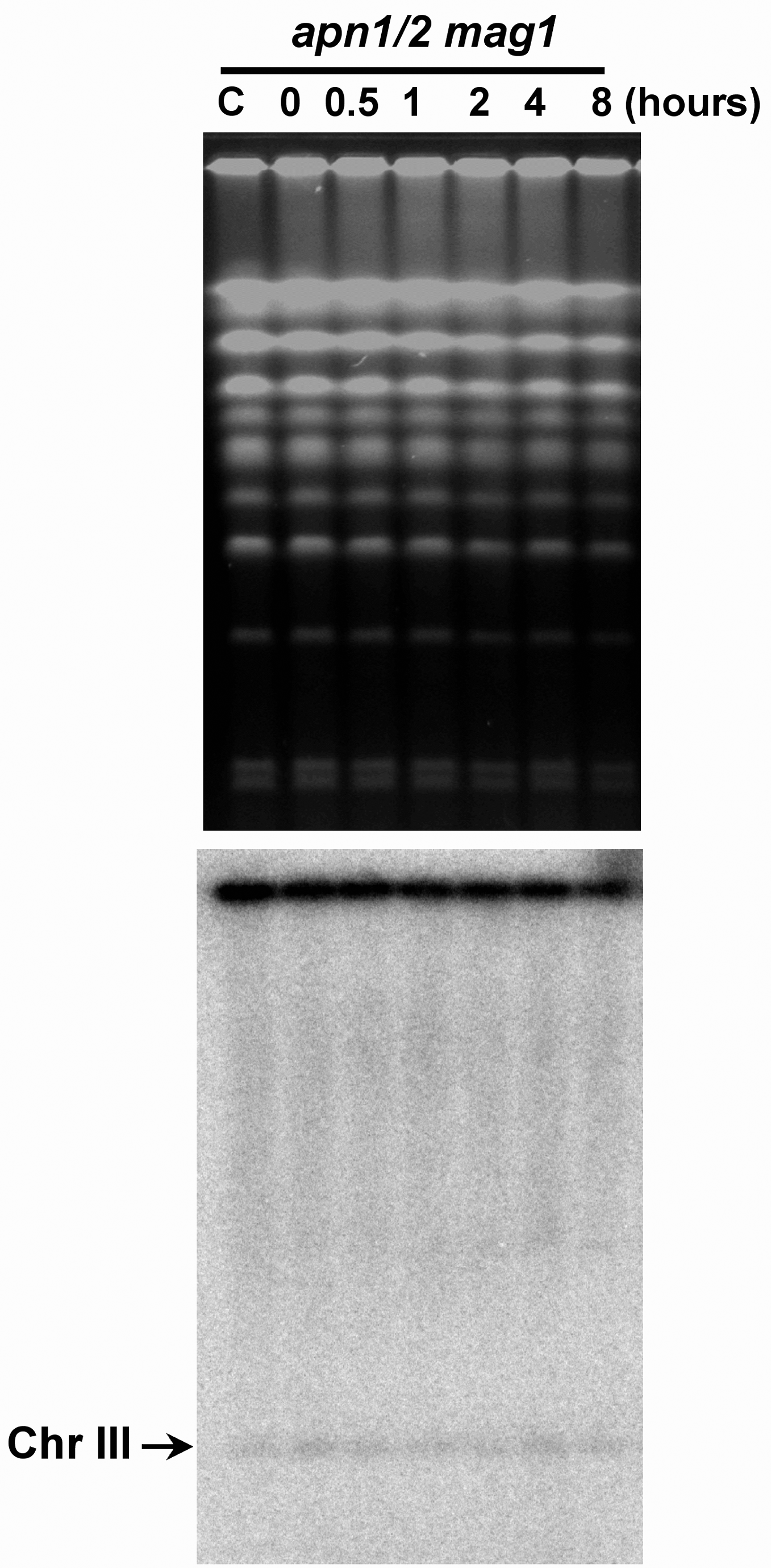

Supplement: Figure S3 — Spontaneous depurination of methylated bases in the absence of MAG1 contributes little to MMS-induced derived DSBs. Logarithmically growing apn1/2 mag1 cells in YPDA were arrested at G2/M by nocodazole, treated with MMS (0.1%, 15 min) in PBS, returned to YPDA+nocodazole and incubated further. Cells were collected at the indicated times and processed for PFGE and Southern blot analysis with the Chr III specific probe CHA1. The induction of DSBs was determined by the appearance of the linear Chr III band. (TIF) [file pgen.1002059.s003.tif]
